# Cross-Spectral Local Descriptors via Quadruplet Network

**DOI:** 10.3390/s17040873

**Published:** 2017-04-15

**Authors:** Cristhian A. Aguilera, Angel D. Sappa, Cristhian Aguilera, Ricardo Toledo

**Affiliations:** 1 Computer Vision Center, Edifici O, Campus UAB, Bellaterra 08193, Barcelona, Spain; asappa@cvc.uab.es (A.D.S.); ricard@cvc.uab.es (R.T.); 2 Computer Science Department, Universitat Autònoma de Barcelona, Campus UAB, Bellaterra 08193, Barcelona, Spain; 3 Facultad de Ingeniería en Electricidad y Computación, CIDIS, Escuela Superior Politécnica del Litoral, ESPOL, Campus Gustavo Galindo, Km 30.5 vía Perimetral, Guayaquil 09-01-5863, Ecuador; 4 DIEE, University of Bío-Bío, Concepción 4051381, Concepción, Chile; cristhia@ubiobio.cl

**Keywords:** cross-spectral, descriptor, infrared, CNN

## Abstract

This paper presents a novel CNN-based architecture, referred to as Q-Net, to learn local feature descriptors that are useful for matching image patches from two different spectral bands. Given correctly matched and non-matching cross-spectral image pairs, a quadruplet network is trained to map input image patches to a common Euclidean space, regardless of the input spectral band. Our approach is inspired by the recent success of triplet networks in the visible spectrum, but adapted for cross-spectral scenarios, where, for each matching pair, there are always two possible non-matching patches: one for each spectrum. Experimental evaluations on a public cross-spectral VIS-NIR dataset shows that the proposed approach improves the state-of-the-art. Moreover, the proposed technique can also be used in mono-spectral settings, obtaining a similar performance to triplet network descriptors, but requiring less training data.

## 1. Introduction

Over the last few years, the number of consumer computer vision applications has increased dramatically. Today, computer vision solutions can be found in video game consoles [1], smartphone applications [2], and even in sports—just to name a few. Furthermore, safety-critical computer vision applications, such as autonomous driving systems, are no longer science fiction.

Ideally, we require the performance of those applications, particularly those that are safety-critical to remain constant under any external environment factors, such as changes in illumination or weather conditions. However, this is not always possible or very difficult to obtain solely using visible imagery, due to the inherent limitations of the images from that spectral band. For that reason, the use of images from different or multiple spectral bands is becoming more appealing. For example, the Microsoft Kinect 2 uses a near-infrared-camera to improve the detection performance in low light or no light conditions.

**Figure 1:**
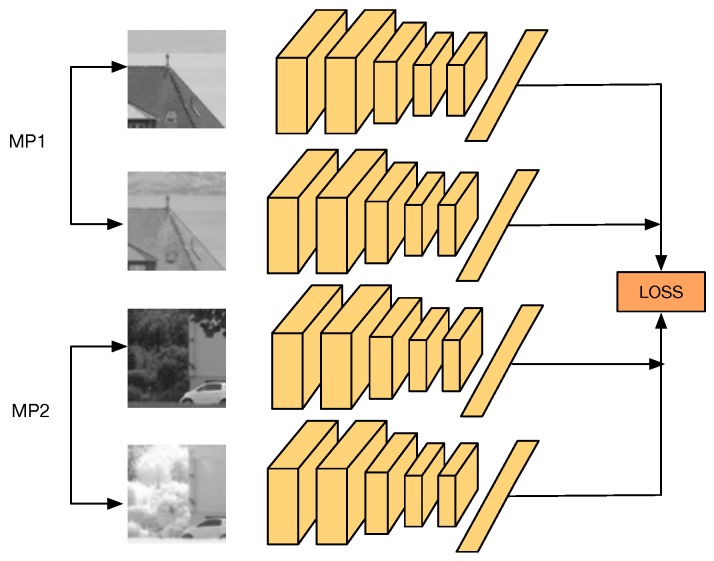
The proposed network architecture. It consists of four copies of the same CNN that accepts as input two different cross-spectral correctly matched image pairs (MP1 and MP2). The network computes the loss based on multiples $L_{2}$ distance comparisons between the output of each CNN, looking for the matching pair with the biggest $L_{2}$ distance and the non-matching pair with the smallest $L_{2}$ distance. Both cases are then used for backpropagation of the network. This can be seen as positive and negative mining.

In this work, we are particularly interested in cross-spectral applications, i.e., computer-vision applications that make use of two different spectral bands to provide a richer representation of a scene, which otherwise could not be obtained from just one spectral band. We present a novel Convolutional Neural Network (CNN) architecture, referred to as Q-Net, to learn local feature descriptors that are useful for matching image patches from two different spectral bands. Figure 1 shows an illustration of our proposal. The training network consists of four copies of the same CNN, i.e., weights are shared, which accepts as input two different cross-spectral image matching pairs (A matching pair consists of two image patches that show the same scene, regardless of the spectral band of the patches. On the contrary, a non-matching pair consists of two image patches that show two different scenes of the world). In the forward pass, the network computes several $L_{2}$ distances between the outputs of each CNN, obtaining the matching pair with the biggest $L_{2}$ distance and the non-matching pair with the smallest $L_{2}$ distance that will be later on used during the backpropagation step. This can be seen as always using the hardest cases of matching and non-matching pairs at each iteration. At testing, our network can be used as drop-in replacements for hand-made feature descriptors such as SIFT [3] in various cross-spectral tasks such as stereo-vision, object detection and image registration. It is important to notice that, during testing, it is just necessary for one of the four CNNs that were used during training. This CNN will act as a feature descriptor.

Our work is based on the recent success of the triplet network presented in [4], named PN-Net, but adapted to work with cross-spectral image pairs, where, for each matching pair, there are two possible non-matching patches—one for each spectrum. Results show that our technique is useful for learning cross-spectral feature descriptors that can be used as drop-in replacements of SIFT-like feature descriptors. Moreover, results also show that our network can be useful for learning local feature descriptors in the visible domain, with similar performance to PN-Net but requiring less training data.

In this article, we make the following contributions:

- We propose and evaluate three ways of using triplets for learning cross-spectral descriptors. Triplet networks were originally designed to work on visible imagery, so the performance on cross-spectral images is unknown.
- We propose a new training CNN-based architecture that outperforms the state-of-the-art in a public Visible and Near-Infrared (VIS-NIR) cross-spectral image pair dataset. Additionally, our experiments show that our network is also useful for learning local feature descriptors in the visible domain.
- Fully trained networks and source code are publicly available at [5].

The rest of the paper is organized as follows. In Section 2, we give a short description of near-infrared images and the VIS-NIR cross-spectral dataset used to train and evaluate our work; differences between visible and infrared images are highlighted. Additionally, Section 2 also presents an overview of hand-made cross-spectral descriptors and CNN-based visible spectrum descriptors. The PN-Net triplet network is described in Section 3, introducing the motivations behind the proposed technique, which are presented in Section 4. Finally, in Section 5, we show the validity of our proposal through several experiments, ending with the conclusions in Section 6.

## 2. Background and Related Work

This section briefly introduces the near-infrared spectral band and highlights similarities and differences between images from this spectrum with respect to the visible spectrum. Additionally, the VIS-NIR cross-spectral image dataset used as a case study through the different sections of this manuscript is also presented. Finally, the most important methods proposed in the literature to describe images from two different spectral bands are reviewed, together with current CNN-based descriptor approaches used to describe images in the visible spectrum.

### 2.1. Near-Infrared Band

**Figure 2:**
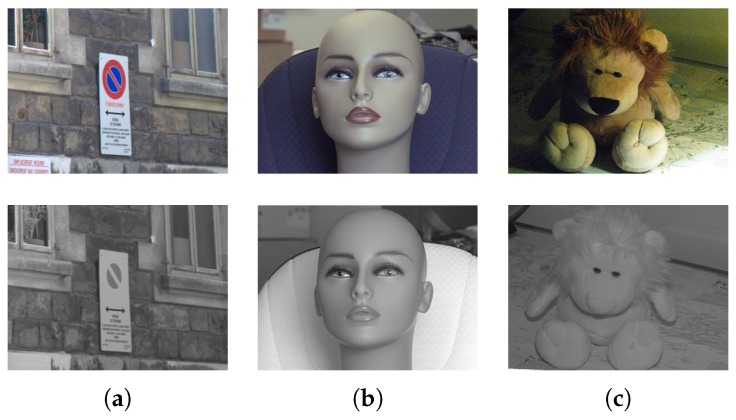
VIS-NIR cross-spectral image pairs; top images are from the visible spectrum and bottom images from the near-infrared spectrum.

The near-infrared band (NIR: 0.7–1.4 μm) is one of the five sub-bands of the infrared spectrum (0.7–1000 μm). It is the closest infrared sub-band to the visible spectrum and images from both spectral bands share several visual similarities; in Figure 2, three pairs of VIS-NIR images are presented. It can be appreciated that images from both spectra are visually similar but with some notable differences. For example, red visible regions disappear in NIR images (see Figure 2a), the direction of the gradients can change (as in Figure 2b), and NIR images are more robust to different illumination settings (as in Figure 2c).

Recent advances in technology have made infrared imaging devices affordable for classical computer vision problems, from face recognition ([6]) to medical imaging ([7]). In some of these cases, infrared images are not used alone but in conjunction with images from other spectral bands (e.g., visible spectra). In these cases, infrared images need to be used in a framework that allows them to be handled in an efficient way in terms of the heterogeneity of the information ([8]), which is the main challenge to be solved and the motivation for current work.

### 2.2. Dataset

**Figure 3:**
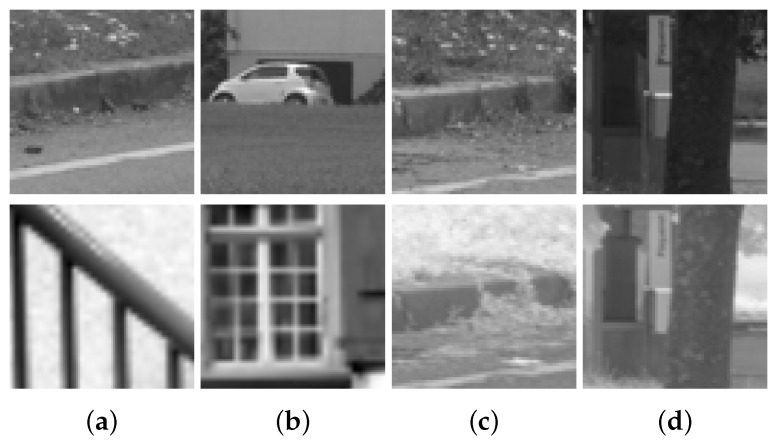
Image patches from the VIS-NIR training set. The first row corresponds to grayscale images from the visible spectrum; and the second row to NIR images. (**a,b**): non-matching pairs; (**c,d**): correctly matched pairs.

The dataset used in [9] has been considered in the current work to train and validate the proposed network. This dataset has been obtained from [10] and consists of more than 1 million VIS-NIR cross-spectral image pairs divided into nine different categories. Table 1 shows the distributions of patches for the different categories. Each image pair consists of two images of 64 × 64 pixels, one NIR and one visible in grayscale format. Finally, the number of matching and non-matching pairs is the same, so half of the samples correspond to correctly matched cross-spectral pairs and the other half to non-matching pairs (Matching and non-matching pairs belong to the same category. The dataset does not contain cross-category pairs). Figure 3 shows four samples of cross-spectral image pairs from the dataset.

### 2.3. Cross-Spectral Descriptors

The description of two images from two different spectral bands in the same way is a challenging task that cannot always be solved with classical feature descriptors such as SIFT ([3]) or SURF ([11]), due to the non-linear intensity variations that may exist between images from two different spectral bands. Early efforts focused on modifying gradient-based descriptors to work between [0,π] instead of [0,2π] to reduce the effect of changes in the gradient direction between images from two different spectral bands. For example, Refs. [12,13] applied this strategy to SIFT and HOG, respectively, the first to match VIS-NIR local features and the second to compute the stereo disparity between a VIS and a thermal infrared camera. Although this strategy is simple, it improves the performance of those algorithms in cross-spectral scenarios.

Other works are based on the observations of [14]. In this study, concerning the joint statistics of visible and thermal images, the authors found a strong correlation between object boundaries of images from both spectra, i.e., texture information is lost and edge information remains similar between images from the different spectral bands. Ref. [15] describes cross-spectral image patches using a local version of the global EHD descriptors, focusing more on the information provided by the edges rather than in image texture. In a similar way, Refs. [16,17] compute cross-spectral features using the EHD algorithm over the image patch response to different Log-Gabor filters.

In a more recent work, Ref. [9] tested different CNN-based networks to measure the similarity between images from the VIS-NIR and the VIS-LWIR spectra. In their experiments, they showed that CNN-based networks can outperform the state-of-the-art in terms of matching performance. However, with regard to speed, their networks are much slower than classical solutions, a problem that we address in the current work, training a cross-spectral descriptor that can be used as a replacement of SIFT and many other $L_{2}$-based descriptors.

It is important to note that many methods that are not based on the use of feature descriptors have been proposed in the literature to match images from different spectral bands. For example, Ref. [18] proposes a non-rigid visible and infrared face registration technique based on the Gaussian field criterion, and Ref. [19] uses a dense matching strategy based on variational approaches to match different multi-modal images.

### 2.4. CNN-Based Feature Descriptor Approaches

During the last decades, carefully *hand-made* feature descriptors, such as SIFT or SURF, have been popular in the computer vision community. However, in the last few years, such approaches have started to be outperformed by CNN-based solutions in different feature descriptors benchmarks (e.g., [4,20,21]). Ref. [20] proposes a max–margin criterion over a siamese network to compute the similarity between two different image patches. Ref. [21] follows a similar approach, but instead of using a metric network, it directly minimizes the $L_{2}$ distance between the descriptor of two images in the loss function, making their trained descriptor a drop-in replacement to SIFT-like descriptors. More importantly, it notices that after a few training epochs, most of the non-matching samples used to train the network were not giving new information, making it necessary to use mining strategies to improve the performance of the networks. In the same way, Ref. [4] proposes a triplet network to mine negative samples with each input triplet, improving the performance of siamese networks.

The current works are strongly based on the triplet network proposed by [4], but adapted to be used in cross-spectral image pairs. We use quadruplets instead of triplets, and we do not only mine non-matching samples but also correctly matched samples. In Section 3 and Section 4, both architectures are detailed.

Notice that, in this review, we only include CNN-based feature descriptors that can be used as a replacement for common *hand-made* descriptors, since they are useful for real-time applications. Other solutions have been proposed skipping the process of description and matching, doing everything with a CNN and trading matching performance for speed—for example, the 2ch network from [20], MatchNet from [22] and the siamese network from [23].

## 3. PN-Net (Triplet Network)

In this section, we describe the architecture of PN-Net ([4]), a state-of-the-art triplet network for learning local features descriptors. Although PN-Net was not intended to be used as a network for learning cross-spectral descriptors, we propose three ways to use PN-Net in such settings. In summary, this section objective is twofold:

- As previously stated, our network is similar to the triplet network but specifically designed to learn cross-spectral local feature descriptors. A brief description of this network will help to set the basis of our proposal in Section 4.
- We explain the motivation behind our proposal through several experiments. After training PN-Net to learn cross-spectral feature descriptors, we discovered that the network performance improved when we randomly alternated between non-matching patches from both spectra.

### 3.1. PN-Net Architecture

**Figure 4:**
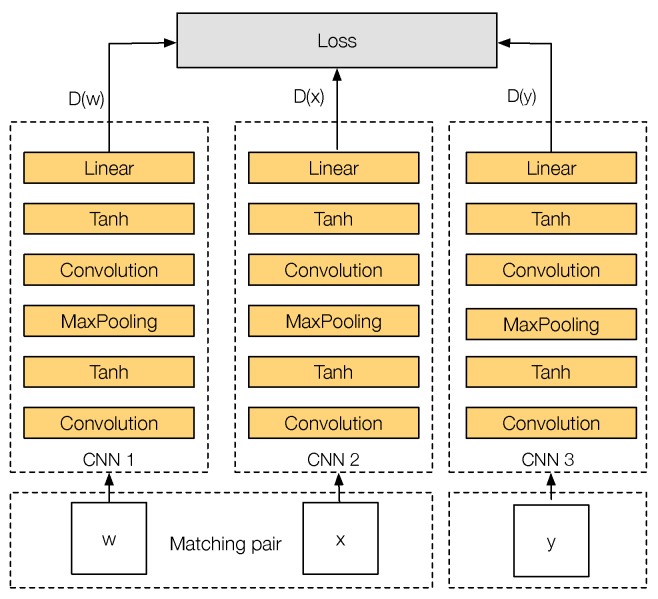
PN-Net training triplet architecture.

Figure 4 shows the training architecture of PN-Net. The network has three inputs, where each input corresponds to a different image patch. Formally, the input is a tuple T={w,x,y}, where *w* and *x* are two matching image patches and *y* is a non-matching image patch to *w* and *x*. Each one of these patches will feed one of the three CNN *towers* that the network has: CNN 1, CNN 2 and CNN 3. The three CNN *towers* of the network share the same parameters during the entire training stage. Finally, the output of each *tower* will be a descriptor *D* of configurable size that describes each input patch.

### 3.2. PN-Net Loss

The loss function is described as follows:

(1) $$\begin{array}{c} P_{m}=\frac{e^{\Delta^{+}}}{e^{min(\Delta_{1}^{-},\Delta_{2}^{-})}+e^{\Delta^{+}}}, \end{array}$$

(2) $$\begin{array}{c} P_{nm}=\frac{e^{min(\Delta_{1}^{-},\Delta_{2}^{-})}}{e^{min(\Delta_{1}^{-},\Delta_{2}^{-})}+e^{\Delta^{+}}}, \end{array}$$

(3) $$\begin{array}{c} Loss\left(T_{i}\right)=P_{m}^{2}+{\left(P_{nm}-1\right)}^{2}, \end{array}$$

where $\Delta^{+}$ corresponds to the $L_{2}$ distance between the descriptors of the matching pair *w* and *x*, ${\left||D\left(w\right),D\left(x\right)|\right|}_{2}$; $\Delta_{1}^{-}$ corresponds to the $L_{2}$ distance between the descriptors of the non-matching pair *w* and *y*, ${\left||D\left(w\right),D\left(y\right)|\right|}_{2}$; and $\Delta_{2}^{-}$ corresponds to the $L_{2}$ distance between the descriptors of the second non-matching pair *x* and *y*, ${\left||D\left(x\right),D\left(y\right)|\right|}_{2}$.

In essence, the objective of the loss function is to penalize small $L_{2}$ distances between non-matching pairs, and large $L_{2}$ distances between matching pairs. Ideally, we want $P_{m}$ to be equal to zero and $P_{nm}$ to be equal to one, i.e, $\Delta^{+}<<min\left(\Delta_{1}^{-},\Delta_{2}^{-}\right)$. Computing the minimum $L_{2}$ distances between the non-matching pairs is a type of mining strategy, were the network always performs backpropagation using the hardest non-matching sample of each triplet *T*, i.e, the non-matching sample with the smallest $L_{2}$ distance. The mining strategy is used to avoid the problems described in [21] and mentioned in Section 2. Finally, the MSE is used to penalize values of $P_{m}$ different than zero and values of $P_{nm}$ different than one.

### 3.3. Cross-Spectral PN-Net

One key difference between mono-spectral and cross-spectral image pairs is that, for each cross-spectral matching pair, we have two non-matching possible image patches—one for each spectrum. Thus, the question is, which image patch do we use as *y*? We propose three simple and naive solutions: (i) *y* is an RGB non-matching image; (ii) *y* is an NIR non-matching image and (iii) *y* is randomly chosen between RGB and NIR.

We test each one of the aforementioned solutions in the dataset used in [9] and presented in Section 2 . We train each network nine times, once per category and tested on the other eight categories. In Table 2, we present our results in terms of the false positive rate at 95% Recall (FPR95). In essence, we evaluate how well the descriptors can distinguish between correct and incorrect matches. Results show that randomly using *y* between an NIR and RGB patch was better than the other two solutions. This gives us a hint that using images from both spectra to mine the network is better than using images just from one, which we expected, assuming that a balanced number of non-matching images from both spectra will help to produce better results.

The motivations behind our quadruplet network are straightforward. As stated before, for each cross-spectral matching pair, we have at least two non-matching patches from another spatial location, each one from one of the spectra to be trained. Similar to triplets, we propose Q-Net, a quadruplet network for learning cross-spectral feature descriptors.

### 3.4. Q-Net Architecture

**Figure 5:**
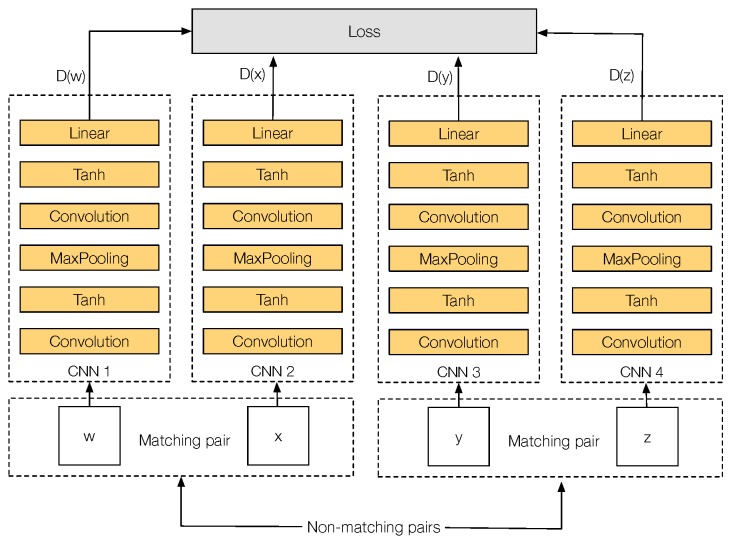
Q-Net training quadruplet architecture.

The architecture of Q-Net is similar to PN-Net, but using four copies of the same network instead of three (see Figure 5). The input is a tuple *Q*, with four different input patches Q={w,x,y,z}, which is formed by two different cross-spectral matching pairs: (*w*, *x*), and (*y*, *z*), allowing the network to mine not just non-matching cross-spectral image pairs at each iteration, but also cross-spectral correctly matched pairs.

## 4. Q-Net

### 4.1. Q-Net Loss

Q-Net loss function extends the mining strategy from PN-Net presented in Section 3.2. Specifically, we add two more distance comparisons to $P_{nm}$, making the loss suitable for cross-spectral scenarios, and we extend the mining strategy from the non-matching pairs to the correctly matched pairs. At each training step, the network uses the matching pair with larger $L_{2}$ distance and the non-matching pair with the smallest $L_{2}$ distance. The loss function is described as follows:

(4) $$\begin{array}{c} P_{m}=\frac{e^{max(\Delta_{1}^{+},\Delta_{2}^{+})}}{e^{min(\Delta_{1}^{-},\Delta_{2}^{-},\Delta_{3}^{-},\Delta_{4}^{-})}+e^{max(\Delta_{1}^{+},\Delta_{2}^{+})}}, \end{array}$$

(5) $$\begin{array}{c} P_{nm}=\frac{e^{min(\Delta_{1}^{-},\Delta_{2}^{-},\Delta_{3}^{-},\Delta_{4}^{-})}}{e^{min(\Delta_{1}^{-},\Delta_{2}^{-},\Delta_{3}^{-},\Delta_{4}^{-})}+e^{max(\Delta_{1}^{+},\Delta_{2}^{+})}}, \end{array}$$

(6) $$\begin{array}{c} Loss\left(Q_{i}\right)=P_{m}^{2}+{\left(P_{nm}-1\right)}^{2}, \end{array}$$

where $\Delta_{1}^{+}$ corresponds to the $L_{2}$ distance between the descriptors of the matching pair *w* and *x*, ${\left||D\left(w\right),D\left(x\right)|\right|}_{2}$; $\Delta_{2}^{+}$ corresponds to the $L_{2}$ distance between the descriptors of the matching pair *y* and *z*, ${\left||D\left(y\right),D\left(z\right)|\right|}_{2}$; $\Delta_{1}^{-}$ corresponds to the $L_{2}$ distance between the descriptors of the non-matching pair *w* and *y*, ${\left||D\left(w\right),D\left(y\right)|\right|}_{2}$; $\Delta_{2}^{-}$ corresponds to the $L_{2}$ distance between the descriptors of the non-matching pair *x* and *y*, ${\left||D\left(x\right),D\left(y\right)|\right|}_{2}$; $\Delta_{3}^{-}$ corresponds to the $L_{2}$ distance between the descriptors of the non-matching pair *w* and *z*, ${\left||D\left(w\right),D\left(z\right)|\right|}_{2}$; and $\Delta_{4}^{-}$ corresponds to the $L_{2}$ distance between the descriptors of the non-matching pair *x* and *z*, ${\left||D\left(x\right),D\left(z\right)|\right|}_{2}$.

The proposed loss function takes into account all the possible non-matching combinations. For example, if we want to train a network to learn similarities between the VIS and the NIR spectral bands, $P_{nm}$ will compare two VIS-NIR non-matching pairs: one VIS-VIS non-matching pair and one NIR-NIR non-matching pair, instead of using a random function as we did with PN-Net. Moreover, since we are trying to learn a common representation between the NIR and the VIS, comparing VIS-VIS and NIR-NIR cases helps the network to have more training examples. Since it is necessary to have two cross-spectral matching pairs to compute $P_{nm}$, it was natural to extend the mining strategy to $P_{m}$, obtaining at each step the cross-spectral matching pair with the larger $L_{2}$ distance.

Our method allows for learning cross-spectral distances, mining positives and negatives samples at the same time. This approach can also be used in monospectral scenarios, providing a more efficient mining strategy than previous works. Results that support our claim are presented in the next section. More importantly, our method can be extended to other cross-spectral or cross-modality scenarios. Even more, it can be extended to other applications such as heterogeneous face recognition, where it is necessary to learn distance metrics between faces from different spectral bands.

## 5. Experimental Evaluation

In this section, we evaluate the performance of the proposed Q-Net on (1) the VIS-NIR dataset introduced in Section 2.2; and (2) the VIS standard benchmark for feature descriptors from [24]. The performance, in both cases, is measured using the FPR95 as in Section 3.

### 5.1. VIS-NIR Scene Dataset

In this section, we evaluate the performance of our network on the VIS-NIR dataset presented in Section 2. As in [9], we train on the country sequence and test in the remaining eight categories.

**Training:** Q-Net and PN-Net networks were trained using Stochastic Gradient Descent (SGD) with a learning rate of 1.1, weight decay of 0.0001, batch size of 128, momentum of 0.9 and learning rate decay of 0.000001. Trained data was shuffled at the beginning of each epoch and each input patch was normalized to its mean intensity. The trained data was split into two, where 95% of the data was used as training data and 5% as validation. Training was performed with and without data augmentation (DA), where the augmented data was obtained by flipping the images vertically and horizontally, and rotating the images by 90, 180 and 270 degrees. Each network was trained ten times to account for randomization effects in the initialization. Lastly, we used a grid search strategy to find the best parameters.

**Figure 6:**
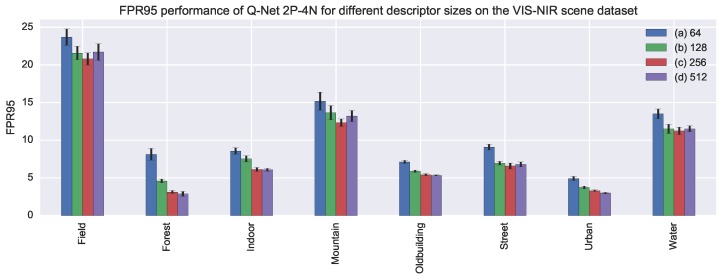
FPR95 performance on the VIS-NIR scene dataset for Q-Net 2P-4N using different descriptor sizes ((**a**) 64; (**b**) 128; (**c**) 256 and (**d**) 512). Shorter bars indicate better performances. On top of the bars, standard deviation values are represented with segments.

**Model details:** Model details are described in Table 3. The layers and parameters are the same from [4], which, after several experimental results, proved to be suitable for describing cross-spectral patches. Notice that, for feature description, shallow models are suitable, since lower layers are more general than the upper ones. The descriptor size was obtained through experimentation. We tested the performance when different descriptor sizes were used. Figure 6 shows the results of our experiment. From the figure we can see that there is a gain in increasing the descriptor size until 256. Descriptor sizes bigger than 256 did not perform better.

**Software and hardware:** All the code was implemented using the Torch framework ([25]). The GPU consisted of an NVIDIA Titan X and the network was trained in between five and ten hours when we used data augmentation.

**Figure 7:**
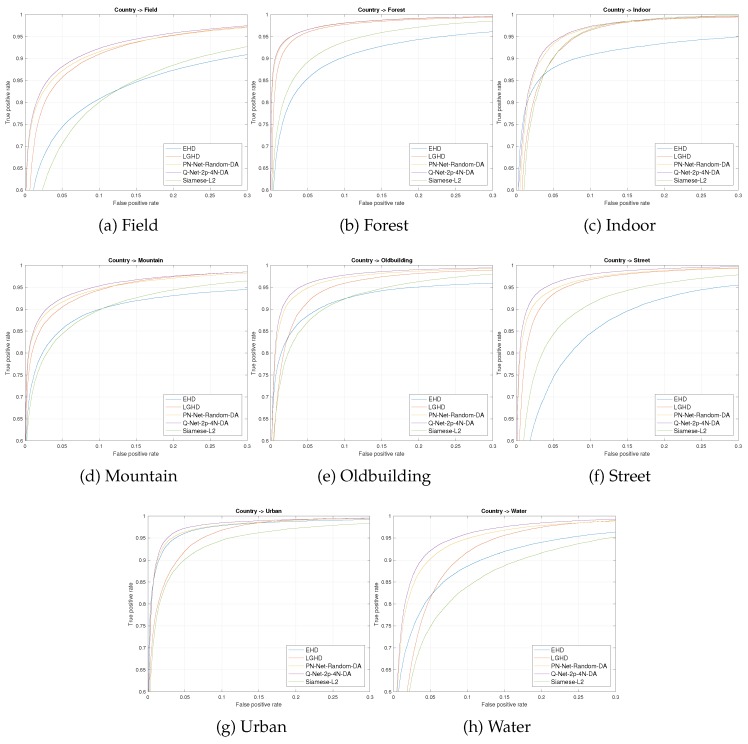
ROC curves for the different descriptors evaluated on the VIS-NIR dataset. For Q-Net and PN-Net, we selected the network with the best performance. Each subfigure shows the result in one of eight tested categories of the dataset.

Results are shown in Table 4. Firstly, we evaluated EHD ([15]) and LGHD ([17]), two *hand-made* descriptors that were used as a baseline in terms of matching performance. The performance of LGHD is under 10% and can be considered as state-of-the-art results before the current work. Secondly, we test a siamese $L_{2}$ network based on the work of [20] that performs better than EHD, but worse than the state-of-the-art. Thirdly, PN-Net and its variant were tested, not being able to surpass the performance of LGHD without using data augmentation. In the other case, Q-Net proved to be better than the state-of-the-art even without data augmentation, showing the importance of mining on the non-matching and matching samples in cross-spectral scenarios. Additionally, we tested our model increasing the training data using the previously detailed data augmentation technique, improving the state-of-the-art by 2.91%. For a more detailed comparison of the different feature descriptors evaluated in the current work, we provide the corresponding Receiver Operating Characteristic (ROC) curves in Figure 7.

In addition, we tested the performance when different descriptor sizes were used. Figure 6 shows the results of our experiment. From the figure, we can see that there is a gain in increasing the descriptor size until 256. Descriptor sizes bigger than 256 did not perform better.

### 5.2. Multi-View Stereo Correspondence Dataset

Although the proposed approach has been motivated to tackle the cross-spectral problem, in this section, we evaluate the proposed architecture when a visible spectrum dataset is considered. This is intended to evaluate the validity of the proposed approach in classical scenarios.

For the evaluation, we used the *multi-view stereo correspondence dataset* from [24], which is considered a standard benchmark for testing local feature descriptors in the visible domain (e.g., [4,20,21,22]). The dataset contains more than 1.2 million patches of size 64 × 64 divided into three different sets: Liberty, Notredame and Yosemite, where each image patch was computed from *Difference of Gaussian* (DOG) maxima. We followed the standard protocol of evaluation, training our network three times, one at each sequence, and testing the FPR95 in the remaining two sequences. In our evaluation, we compared our model against two other learned $L_{2}$ descriptors, the first from [20] and the second from [4]; which can be considered state-of-the-art.

**Training**. Quadruplet networks were trained using Stochastic Gradient Descent (SGD) with a learning rate of 0.1, weight decay of 0.0001, batch size of 128, momentum of 0.9 and learning rate decay of 0.000001. Trained data was shuffled at the beginning of each epoch and each input patch was normalized using zero-mean and unit variance. We split up each training sequence into two sets, where 80% of the data was used as training data and the 20% left as validation data. We used the same software and hardware from the previous experiment. As in the previous experiment, Q-Net and PN-Net networks were trained ten times to account for randomization effects in the initialization.

Table 5 shows the results of our experiments. Q-Net and PN-Net performed better than the siamese-L2 network proposed by [20], which is an expected result, since the siamese-L2 network was not optimized for $L_{2}$ comparison during training as the other two networks were. Q-Net performed better than PN-Net by a small margin but using much less training data. When comparing both techniques with the same amount of data, the difference becomes bigger—meaning that our network needs less data to train than PN-Net, i.e., Q-Net needs less training data than PN-Net and it converges more quickly.

Regarding training time, both networks perform similarly. In our experiments, PN-Net was about 9% faster than Q-Net when both networks where trained with the same amount of patches. In essence, the improved accuracy performance of Q-Net is related to a small loss in training speed.

## 6. Conclusions

This paper presents a novel CNN-based architecture to learn cross-spectral local feature descriptors. Experimental results with a VIS-NIR dataset showed the validity of the proposed approach, improving the state-of-the-art by almost 3%. The experimental results showed that the proposed approach is also valid for training local feature descriptors in the visible spectrum, providing a network with similar performance to the state-of-the-art, but requiring less training data.

Future work might consider using the same architecture for different cross-spectral applications such as heterogeneous face recognition.

## Figures and Tables

**Table 1:** Shows the number of cross-spectral image pairs per category on the VIS-NIR patch dataset used to train and evaluate our work.

Category	# Cross-Spectral Pairs
country	277,504
field	240,896
forest	376,832
indoor	60,672
mountain	151,296
old building	101,376
street	164,608
urban	147,712
water	143,104

**Table 2:** Average FPR95 for each category.

Train seq.	PN-Net Gray	PN-Net NIR	PN-Net Random
country	11.79	11.63	**10.65**
field	17.84	16.56	**16.10**
forest	36.00	32.47	**32.19**
indoor	48.21	47.26	**46.48**
mountain	29.35	26.29	**25.67**
old building	29.22	**27.25**	27.69
street	18.23	**16.71**	16.73
urban	**32.78**	36.61	33.35
water	18.16	17.76	**15.84**
average	26.84	25.84	**25.08**

**Table 3:** Q-Net layer descriptions.

Layer	Description	Kernel	Output Dim
1	Convolution	7 × 7	32 × 26 × 26
2	Tanh	-	32 × 26 × 26
3	MaxPooling	2 × 2	32 × 13 × 13
4	Convolution	6 × 6	64 × 8 × 8
5	Tanh	-	64 × 8 × 8
6	Linear	-	256

**Table 4:** FPR95 performance on the VIS-NIR scene dataset. Each network, i.e., siamese-L2, PN-Net and Q-Net, were trained in the country sequence and tested in the other eight sequences as in [9]. Smaller results indicate better performance. In brackets, the standard deviation is provided.

Descriptor/Network	Field	Forest	Indoor	Mountain	Old Building	Street	Urban	Water	Mean
EHD	48.62	23.17	30.25	33.94	19.62	27.29	3.72	23.46	26.26
LGHD	18.80	3.73	8.16	11.34	8.17	6.66	7.39	13.90	9.77
siamese-L2	38.47	12.46	7.94	22.36	15.70	16.85	11.06	29.18	15.50
PN-Net RGB	$\underset{\left(1.08\right)}{25.33}$	$\underset{(0.28}{4.41}$	$\underset{\left(0.32\right)}{7.00}$	$\underset{\left(1.07\right)}{19.37}$	$\underset{\left(0.32\right)}{7.31}$	$\underset{\left(0.46\right)}{10.21}$	$\underset{\left(0.27\right)}{5.00}$	$\underset{\left(0.67\right)}{17.79}$	$\underset{\left(0.40\right)}{12.05}$
PN-Net NIR	$\underset{\left(0.98\right)}{24.74}$	$\underset{\left(0.14\right)}{4.45}$	$\underset{\left(0.25\right)}{6.54}$	$\underset{\left(0.44\right)}{15.75}$	$\underset{\left(0.19\right)}{7.78}$	$\underset{\left(0.25\right)}{10.82}$	$\underset{\left(0.14\right)}{4.66}$	$\underset{\left(0.34\right)}{16.49}$	$\underset{\left(0.15\right)}{11.40}$
PN-Net Random	$\underset{\left(1.00\right)}{24.56}$	$\underset{\left(0.20\right)}{3.91}$	$\underset{\left(0.43\right)}{6.56}$	$\underset{\left(0.60\right)}{15.99}$	$\underset{\left(0.31\right)}{6.84}$	$\underset{\left(0.36\right)}{9.51}$	$\underset{\left(0.34\right)}{4.407}$	$\underset{\left(0.61\right)}{15.62}$	$\underset{\left(0.34\right)}{10.92}$
Q-Net 2P-4N (ours)	$\underset{\left(0.81\right)}{20.80}$	$\underset{\left(0.20\right)}{3.12}$	$\underset{\left(0.27\right)}{6.11}$	$\underset{\left(0.49\right)}{12.32}$	$\underset{\left(0.13\right)}{5.42}$	$\underset{\left(0.40\right)}{6.57}$	$\underset{\left(0.11\right)}{3.30}$	$\underset{\left(0.50\right)}{11.24}$	$\underset{\left(0.14\right)}{8.61}$
PN-Net Random DA	$\underset{\left(0.65\right)}{20.09}$	$\underset{\left(0.27\right)}{3.27}$	$\underset{\left(0.14\right)}{6.36}$	$\underset{\left(0.57\right)}{11.53}$	$\underset{\left(0.20\right)}{5.19}$	$\underset{\left(0.20\right)}{5.62}$	$\underset{\left(0.28\right)}{3.31}$	$\underset{\left(0.36\right)}{10.72}$	$\underset{\left(0.24\right)}{8.26}$
Q-Net 2P-4N DA (ours)	$\underset{\left(0.33\right)}{17.01}$	$\underset{\left(0.17\right)}{2.70}$	$\underset{\left(0.18\right)}{6.16}$	$\underset{\left(0.38\right)}{9.61}$	$\underset{\left(0.18\right)}{4.61}$	$\underset{\left(0.09\right)}{3.99}$	$\underset{\left(0.13\right)}{2.83}$	$\underset{\left(0.14\right)}{8.44}$	$\underset{\left(0.09\right)}{6.86}$

**Table 5:** Matching results in the *multi-view stereo correspondence dataset*. Evaluations were made on the 100 K image pairs’ ground truth recommended from the authors. Results correspond to FPR95. The smallest results indicate better performance. The standard deviation is provided in brackets.

Training	Notredame	Liberty	Notredame	Yosemite	Yosemite	Liberty	
Testing	Yosemite		Liberty		Notredame		
Descriptor							mean
siamese-L2	15.15	20.09	12.46	8.38	18.83	6.04	13.49
$\underset{\mathrm{size}\;=\;128,\;\mathrm{patches}\;=\;2,560,000}{\text{PN-Net}}$	$\underset{\left(0.20\right)}{8.47}$	$\underset{\left(0.48\right)}{9.50}$	$\underset{\left(0.17\right)}{9.17}$	$\underset{\left(0.49\right)}{10.82}$	$\underset{\left(0.18\right)}{4.47}$	$\underset{\left(0.10\right)}{4.16}$	$\underset{\left(0.17\right)}{7.77}$
$\underset{\mathrm{size}\;=\;128,\;\mathrm{patches}\;=\;3,840,000}{\text{PN-Net}}$	$\underset{\left(0.46\right)}{8.46}$	$\underset{\left(0.23\right)}{8.77}$	$\underset{\left(0.11\right)}{8.86}$	$\underset{\left(0.57\right)}{10.78}$	$\underset{\left(0.14\right)}{4.37}$	$\underset{\left(0.10\right)}{3.98}$	$\underset{\left(0.16\right)}{7.53}$
$\underset{\mathrm{size}\;=\;128,\;\mathrm{patches}\;=\;2,560,000}{\text{Q-Net}\;\text{2P-4N}}$	$\underset{\left(0.52\right)}{7.69}$	$\underset{\left(0.71\right)}{9.34}$	$\underset{\left(0.31\right)}{7.64}$	$\underset{\left(0.60\right)}{10.22}$	$\underset{\left(0.18\right)}{4.07}$	$\underset{\left(0.13\right)}{3.76}$	$\underset{\left(0.22\right)}{7.12}$
